# Spinal Affections, and the Prone System of Treating Them

**Published:** 1846-01

**Authors:** 


### Spinal Affections, and the Prone System of treating them; being an Enquiry into the Nature, Causes, and different Methods of treating Diseases and Distortions of the Spinal Column, &c. By *James Coles,* M.R.C.S.E. 12mo, pp. 320. London, 1845. Houlston and Stoneman.

The subject of Spinal Deformities is one that demands the very serious attention of medical men, not less for the importance of the malady involved, than for the great discrepancy of opinion that has too long (it must be confessed) prevailed as to the best method of its treatment. The publication of the present volume gives us the opportunity of bringing the matter under the notice of our readers, and of making them acquainted with the most recent observations that are calculated to throw light upon it. Without further preface we shall at once introduce Mr. Coles to their acquaintance.

In the following passage, he gives a lucid description of the (often) obscure symptoms in the incipient stage of a Lateral Curvature of the Spine.

"The attention of the mother, or governess, of a young girl who is becoming the subject of lateral curvature of the spine, is first attracted by the appearance of inequality, either in the shoulder or hips; and this inequality is more readily detected, if either party has been absent from the other for a short period. It is likewise not uncommon for the attention of a parent to be first drawn to the circumstance by a friend, or casual visitor. This inequality consists not, at this time, of the one shoulder being elevated above the other, but of its appearing to be thickened and larger, and in older girls this unequal appearance is also conspicuous in the breast of one side, generally the right.

"Again, it sometimes happens, that an irregular and unsteady mode of walking first excites attention; the ankle on one side seems weaker, and an appearance of limping in the carriage is observable. When the patient runs or walks fast, the limb of the affected side in being brought forward, is thrown outwards, and swung in advance, as it were, with a jerking effort not easily described, but readily detected by a person previously acquainted with the symptom. This affection of the leg, which is the result of inequality at the hips, is apparently so little connected with the vertebral column, that I have often witnessed the incredulous smile of friends, when, on being consulted for this supposed weakness of the ankle, I have requested to be allowed to examine the back of the patient.

"On doing so at this stage of the disease, and tracing the vertebral column, with ink, from the neck to the loins, the distance of the two shoulder-blades, from the line so marked, will be found unequal; the right, towards which the column in the majority of cases curves, being nearer, by from one-eighth of an inch to half an inch, than the left; and if in addition a plumb line be applied to the spine, this inequality will be found to arise from the curvature of the column, and not, as was previously supposed, from a growing out of the shoulder. At this early period the only other conspicuous appearance in the back will be a slight sinking in of the right loin, and a proportionate fullness of the opposite side; and in cases where the peculiarity in walking above described exists, a slight elevation of the hip on the left side."* P. 38.

---

* The diagnosis will be much assisted, if the patient be made to bend her body well forwards, the arms being crossed upon the chest, "until the spine is

The constitutional health is generally delicate, and, in very many cases, the growth of the body is unusually rapid, the girl shooting up in height like a tender sapling that has not strength to keep it upright. She is often blamed for stooping or sitting awry; whereas these positions are the immediate, and almost necessary, results of the spinal weakness and irregularity. Mr. Coles, like every judicious practitioner, deprecates the use of immoderate exercise, either in or out of doors, in such cases. The general health must be invigorated, and the patient should rest a great deal in the recumbent posture; the amount of exercise, to be taken at proper intervals, being regulated according to the circumstances of each individual case.

If the case has been neglected or mismanaged, the spinal deformity will become aggravated in the following way :—

" The spine now begins to assume not only a curved but also a rotated form, having in fact become twisted upon its axis; the attachments of the ribs on the right side are thus carried outwards and backwards; the ribs themselves are compressed and bent, and thus made to form a projecting ridge posteriorly, sometimes of considerable magnitude, and adding greatly to the appearance of deformity; on the opposite side, the attachments of the ribs to the spine are carried inwards and towards the right, the whole of the left side is consequently depressed, and flattened; and the shoulder-blade accompanying the ribs, is drawn below the level of its fellow, and is more or less displaced. As the disease progresses, and the debility increases, the distorted column begins to project backward as well as laterally, bringing with it the right shoulder and the projecting ridge of ribs, the body tilts over to the left side, which becomes so sunken, shrivelled, and depressed, that the ribs are not unfrequently found to rest upon the ascending hip, and the deformity becomes complete." P. 45.

The chapter on the predisposing and exciting Causes of spinal Curvature contains many valuable and judicious observations. Mr. Coles very properly traces the deformity in almost every case to constitutional debility, the ill effects of which are too often most wilfully aggravated by fashionable absurdities in the management of dress, by overstrained education, and so forth. We cannot however agree with him in supposing that a genuine *strumous* diathesis exists in all, or even in the majority of, instances; unless, indeed, we consent with M. Lugol to apply that term to all cases of constitutional weakness.

---

brought into a considerable curve, and the skin becomes tightly drawn over the prominent spinous processes, like that over the knuckles of the closed fist; by this attitude the column will be made straight longitudinally, even if a considerable curve exists when the body is erect. Each of the projecting points throughout the column should now be marked with ink, or charcoal, indigo, or any other convenient colouring matter, and the patient again directed to stand upright; if any curve exists it will be now apparent, by the marks, which still form a straight line down the back, not being found in their places over each of the spinous processes of the vertebræ, and this will be still more minutely traceable in difficult cases, by having recourse to a piece of small twine or thread, and placing one end of it on the prominent bone which is situated at the bottom of the neck, and the other on the lowest vertebræ of the loins, where it joins the pelvis; this, by connecting the perpendicular line of spots made by the ink, will show the minutest deviation even of one single bone in the vertebral column."

John Hunter pithily remarked, "I am convinced that people get awry by the endeavours of parents to keep them straight." Certainly nothing has tended more to increase spinal deformities in modern times than the absurd and pernicious use of tight stays, and all those other artificial means that have been devised by fraud or by folly to improve the figure of young females.

"There is another remark," says our author, "of Hunter's, which, properly understood, is replete with valuable instruction, and which applies to an error more prevalent at present than any other in the physical management of youth; I mean the habit which many parents and teachers have acquired of watching every movement and attitude of their young charges, with a view to correct every inelegant or unstudied position of the body.

'If it be necessary from fashion, and so on, to carry the person in any particular manner, this habit may be attained at any period of life. . . . . . . . . . You see a ploughboy, whilst plodding at the plough, an awkward fellow; but he enlists, is placed under a drill sergeant, and then observe how altered in every respect he becomes.'" P. 104.

Mr. Coles is no friend to "those senseless and dangerous substitutes of the present day for the elegant and healthful exercises of a less theoretical age, the modern gymnastics: these have superseded for a time (and it will be only for a time) the practice of dancing and fencing, which tended to produce in our forefathers that superiority in real grace of deportment, which is generally acknowledged to have belonged to the last century."

In the treatment of Spinal Distortion, there are three points that should invariably be kept in view—1, to relieve the affected part of the spine from the superincumbent weight of the head and shoulders; 2, to remove any active disease in this part (if such be present), and afterwards strengthen and invigorate it; and 3, to attend to the constitutional health of the patient. There can be little difference of opinion among professional men not only as to the propriety, but also as to the means to be adopted for the purpose, of effecting the two latter objects. Probably, too, all will agree that the first-named indication is of primary importance; but, hitherto certainly, there has not been much accordance among medical writers as to the best mode of accomplishing it in practice.

M. Coles disapproves of all sorts of patent stays and other instruments that have been recommended as artificial supporters of the spine, such as Chesher's collar, Tavernier's spring-belt, Marshall Hall's electrotyped copper stays, back-boards, shoulder-straps, straight-backed chairs, &c. He very justly remarks:

"If the nature and causes of distortion of the spine, be those which I have previously endeavoured to prove them to be; if, in fact, muscular debility has any connection with the disorder, then, as a general principle, all artificial means of supporting the back, which remove from the muscles their natural stimulus to action, whereby alone they can recover their vigour, must be injurious; and when, combined with the suspension of muscular action, the parts are subjected to considerable and long-continued pressure, as must always be the case from the use of steel stays, and most other spinal supports, the injurious effects are very considerably augmented.

"It is a law of the animal economy, that the exercise of an organ is necessary, not only to its perfection but to its preservation; as also, that if the various parts of the body are not kept in a due state of activity, they degenerate and waste away until they lose their natural appearance and character, and become altered even in structure." P. 118.

The only safe and effectual mode of taking off the superincumbent weight of the head and shoulders from the distorted spine is to have recourse to a judicious and regulated employment of the recumbent position;—but not, as hitherto has been too much the case, by confining a patient for months and even years to the supine posture, upon a hard inclined board, condemned all the while to utter inactivity.

The main object of our author's work is to shew the superior advantages of the *prone* over the *supine* position, in the treatment of spinal deformities.* The objections to the latter are manifold and serious. 1. The injurious effects of pressure, not only upon the integuments covering the sacrum, trochanters, and other projecting bones, but also upon the bones themselves, at least in those cases where the distortion consists in a posterior or angular projection of the spine, are well known to every one. The continued pressure upon the muscles also cannot fail to interfere very seriously with their restoration to health and power: nay, it may even lead to a very considerable absorption of their substance; and, as the flexors of the body are naturally more powerful than the extensors, how much mischief may be caused by the slightest lesion of this sort, we need scarcely say. We cannot therefore be much surprised that a confirmed stoop is a very frequent result of the prolonged use of the reclining board. 2. The continued pressure of the thoracic viscera upon the spinal and sympathetic nerves, and upon the great blood-vessels† that lie upon the anterior surface of the vertebral column, can scarcely fail to produce a certain amount of disturbance in the functions of innervation and circulation. Hence probably the headaches, difficulty of breathing, palpitations of the heart, indigestion, obstinate constipation, weakness of the bladder, hæmorrhoids, varicose state of the veins and œdema of the legs, &c., which are not unfrequently observed in patients who have been long confined to the horizontal supine position. Besides these and similar ailments, such invalids often experience a marked impairment of vision, in consequence of the manner in which the rays of light fall upon the eye, and of their reflection from the white ceiling of the room, upon which the eye must necessarily rest during the greater part of the time spent upon the plane.

---

* To Mr. Bampfield, in his prize essay on Curvatures and Diseases of the Spine, published in 1824, is unquestionably due the merit of having first publicly recommended the use of the prone position in preference to the supine. Dr. Verral may indeed have employed it, in an improved form, a year sooner in his private practice; but there is no evidence to shew that he made any public notice of it for six or seven years afterwards. It is therefore positively absurd in Mr. Coles to claim for his friend "the priority and originality of the invention."

† Mr. Liston, in his recent course of lectures on the diseases of the bones and joints, published in the Lancet, thus speaks of the treatment of spinal diseases. "In the first place, the patient should be kept perfectly quiet, so that the growing together of the parts may be encouraged. *Perhaps the prone position, about which so much has been said lately, is the most favourable*, as it takes the pressure off the diseased parts, and prevents the carious bodies from falling in upon one another. *It also assists the return of the blood from the numerous veins contained in the bodies of the vertebræ, and in the spinal canal.*" P. 162.

Among the leading advantages of the prone position (upon a properly-constructed couch, "carefully fitted to the patient's size and admitting of easy variation in its angles"), we may mention the facility of access to the distorted or diseased parts, for the purposes of remedial applications, friction, and so forth; and the steady and constant extension of the spine that is maintained by the mere weight or dependence of the lower extremities, (resting on the sloping position of the couch), which, it will be obvious, may be kept flexed at the hips to a greater or less degree, according as the circumstances of each case may require.

"The effect of this gentle and continued extension is most beneficial. In cases of curvature only, it materially assists in straightening the back and correcting the deformity, but in all cases of active disease it is of the greatest benefit and importance. In caries of the bones of the spine and ulceration of the cartilages, its effect being to separate the diseased surfaces, and to counteract the pressure which even the contraction of the muscles occasions and renders injurious, one principal cause of absorption of bone and increase of deformity is removed, inflammatory action is allayed, and the relief thus obtained is soon manifested by a subsidence of the most distressing symptoms—pain, a tendency to paralysis of the lower extremities, and increase of the projection." P. 195.

Mr. Coles assures us that several patients affected, with *paralysis* (especially when occasioned by external injury or by irregularities of the spine), have regained the use of their limbs by the simple employment of the prone couch; the extension of the vertebræ, thereby produced, having the effect of relieving the spinal marrow and its nerves from all injurious pressure which may be present.

The prone position is well suited for the treatment of *Psoas Abscess* also. "In the early stage of this disease, while it may be hoped that active remedial measures might be the means of preventing the formation of matter, it is clear that in no other position can the patient be so advantageously placed, as well on account of the perfect quiet that it affords, as the extraordinary facility which it gives for the application of the various remedies. In the incipient stage of the disease of the vertebral column, the prone position is still more imperatively demanded; and, in that case, as well as in inflammation or injuries of the spinal marrow, or of its investing membranes, the long continued and most perfect quietude in which the patient may remain, is assuredly a most important recommendation." When pus has formed and pointed in the groin, it will obviously find a much more ready exit in the prone than in the supine position of the body. In many cases, too, of *Hip-joint Disease*, the greatest comfort and relief will often be experienced by the patient lying upon his face; there is moreover the advantage of the affected part being easily reached for the application of remedies.

In that species of Deformity of the Chest, which consists in a flattening and depression of the ribs laterally, and in an unusual projection of the sternum, the use of the prone couch, along with such exercises as serve to strengthen the muscles that extend from the arms and shoulders to the chest, will be found to be much more simple and efficacious than the employment of direct pressure with the hand upon the projecting portion of the chest, as recommended by Dupuytren.

One great advantage of the prone position is that the patient can not only amuse himself with reading, writing, drawing, and such like studies, but may also practise various exercises with both his upper and lower extremities—exercises that will have a most beneficial effect in strengthening the muscles of the back, and in correcting the spinal irregularity. The appetite and digestion, too, are usually much better in patients treated in this manner than in those who are kept for a length of time lying upon their backs ; the moderate stimulus produced by gentle pressure upon the stomach, and the more ready exit of the food into the bowels, appearing to act beneficially in this respect.

Mr. Coles very properly condemns all merely mechanical means of producing *extension of the back* in cases of spinal distortion. The only safe and rational method of obtaining this result (when no active disease exists in any part of the vertebral column) is through the medium of the muscles themselves; as, for example, by having recourse to the following expedient.

"In front of a common prone couch is placed a small table; round the waist of the patient, whilst reclining on the couch, is fastened a belt, from which a cord on each side passes downwards and backwards to the bottom of the couch, then, passing over a pulley on each side, they return horizontally to the front legs of the table, between which a cross bar stretches on a level with the pulleys at the bottom of the couch; here, passing over other pulleys, they ascend to the top of the table; another pair of pulleys enable the cords to pass that angle, and they then terminate by being inserted into a turned handle of hard wood, like a large ruler, of the required length; the patient grasping this handle at the utmost stretch of her arms, the cords are to be tightened to the necessary degree at the belt round the waist, and the patient then directed to pull herself forward by means of the handle; as she does so, of course, she is held back by the belt round the waist, the effort puts all the muscles of the shoulders, ribs, and chest, into powerful contraction, and the result required is obtained." P. 229.

A more simple plan consists in the patient, on the prone couch, grasping the edge of a table sufficiently wide to oblige her to extend her arms to their full length, the cords for the waist-belt being made fast to some fixed point behind her, either of the door or wall, against which the bottom of the couch must also press ; she then draws herself forward by the arms as before.

When the curve is situated high up in the vertebral column, or in the neck, extension may be effected by the use of a head-swing.

"A padded strap passes under the chin and round the back of the head, the ends meeting just above the temples, from them a strap on each side passes about six inches above the head, where they unite and terminate in a hook; this is fastened, when required to be used, over the loop of a double cord, which passes upwards to a beam or door-way, and over two pulleys fixed in the wood-work; these cords then again descend and terminate in small handles, just within the reach of the extended arms of the patient, she grasps them, and by pulling downwards raises herself off the ground by the head and arms, thus combining both active and passive extension in one operation." P. 231.

In the selection of the Exercises to be recommended for the treatment of lateral curvature in its early stage,—provided always there be no existing local irritation—our author judiciously insists upon the importance of the principle or general rule that the muscles of the back and loins should be brought into active and steady, but not fatiguing, play.

Swinging by the hands—using a skipping-rope, always "backwards," as girls term it—bending the body backward and forward over the end of a sofa or the back of a low chair—sham-rowing—raising a weight attached to a rope which is made to pass over two pulleys, one in the floor and the other in the ceiling*—walking with a weight upon the head, or suspended in front of the body by a strap passed round the shoulders or occiput—these, and other such-like exercises may unquestionably be productive of very decided benefit. "In cases where the unequal action of the muscles of the back require that those on one side should undergo a more vigorous discipline than those of the other, the means for its accomplishment are equally simple, and within the reach of ordinary application. Of these, turning a wheel, either with the right or left arm as the case may be; raising the weight of the pulley with one hand; drawing the cord to which it is attached horizontally from a pulley in the wall of the room, with the hand of the debilitated side; are some of the most effective."

We need scarcely say that, along with the employment of the means to which we have been alluding, the general health must be strengthened by

---

* Mr. Coles very justly disapproves of the too common practice of having only one pulley, and that in the ceiling; the exercise then being to raise the weight from the ground by pulling the handle, affixed to the loose end of the rope, downwards. He explains the very different effects of these two modes of exercise by the following illustration. "One occupation peculiarly exemplifies the effects of the unequal employment of the different classes of muscles, and some of my most effective means of applying local exercise for the cure of deformity, were constructed in accordance with the principles which are here illustrated, and after examination of numerous examples with one uniform result. The two men employed at a saw pit would, at first sight, appear to be doing much the same sort of work, and in much the same kind of manner; and perhaps my readers may feel some surprise at being, for the first time, told that no two occupations can be more opposite in their effect upon the muscles of the back, than that of the man who works above the piece of timber to be cut, and that of the labourer in the pit. Such is the fact, however; the former exerts all his strength in lifting the heavy saw, and throws its handle, with a kind of flourish, high above his head, to the extreme reach of his arms; this he does by a vigorous exertion of all the muscles of the back and loins, and the attitude of a fine man at the moment when his arms are thus elevated, his body thrown back, his legs perfectly straight, and his feet close to each other, whilst his head is slightly bent forward, and his eyes fixed upon the line of chalk at his feet, is one of extreme grace and manly vigour. The effect of such an employment is to communicate a portion of its freedom and grace to the general carriage of the artizan, and 'top-sawyer' has become a proverbialism for designating a fine smart manly-looking young fellow. The occupation of his companion in the pit is of a different character, his active duty commences at the moment when the other's ceases; and the saw being elevated as high as it will go, he applies all his strength to perfect the cut which results from its descent, and to pull down the instrument, which his mate has pulled up, as low as it will go. In doing this, the principal muscles engaged are those situated on the front of the body, or the chest, abdomen, and thighs; in fact, the antagonists of those employed by the top sawyer. The result produced is as opposite as the means which produce them. The pitman acquires a round-shouldered stooping gait, and may easily be distinguished from his fellow by these peculiarities." P. 250.

inhaling a pure air, the use of a nourishing diet, and the exhibition of various tonic medicines, more especially of *steel;* to which small doses of *iodine* may be advantageously added, whenever there is reason to suspect the existence of a scrofulous taint in the constitution.* For further particulars we must refer our readers to Mr. Coles' work: it is written in a candid and unassuming spirit, and will be found to contain many most judicious observations on the subject of which it professes to treat. The only drawbacks to this expression of our praise are that he alludes to an apparatus which he has invented for the benefit of his patients, but which he omits to describe; and that, in the narration of some of his cases, there is a very unnecessary introduction of the name of the Queen Dowager, Lady Elizabeth Tufton and so forth, as recommending patients to the Verral Charity, of which he is the surgeon. One lady writes to him in the most affectionate manner that "the most happy circumstance in my life was the conversation I had with the dear kind friend, who first made me known to the prone couch, and the excellent system you practise in connection with it." This sort of trash should be avoided in every respectable professional work.

* The tincture of Iodine will be found a useful outward application in several forms of spinal disease.

